# Preservative Effect on Canned Mackerel (*Scomber colias*) Lipids by Addition of Octopus (*Octopus vulgaris*) Cooking Liquor in the Packaging Medium

**DOI:** 10.3390/molecules27030739

**Published:** 2022-01-24

**Authors:** José M. Malga, Marcos Trigo, Beatriz Martínez, Santiago P. Aubourg

**Affiliations:** 1 Department of Food Technology, Marine Research Institute (CSIC), c/Eduardo Cabello, 6, 36208 Vigo, Spain; jmalga@alumnos.uvigo.es (J.M.M.); mtrigo@iim.csic.es (M.T.); 2 Department of Food Technologies, CIFP Coroso, Avda. da Coruña, 174, 15960 Ribeira, Spain; bmartinezr@edu.xunta.gal

**Keywords:** *octopus vulgaris*, cooking liquor, *scomber colias*, canning, packaging medium, lipid hydrolysis, lipid oxidation, polyene index, ω3/ω6 ratio

## Abstract

The preservative properties of waste liquor obtained from octopus (*Octopus vulgaris*) cooking were investigated. Three different concentrations (high, medium, and low) of octopus cooking liquor (OCL) were included, respectively, in the aqueous packaging medium employed for mackerel (*Scomber colias*) canning. As a result, the canning process led to an increase (*p* < 0.05) of lipid content, lipid oxidation (development of fluorescent compounds and thiobarbituric acid reactive substances, TBARS), lipid hydrolysis (formation of free fatty acids, FFA) and ω3/ω6 ratio in fish muscle. In all canned samples, primary (peroxides) and secondary (TBARS) levels of lipid oxidation were low. Remarkably, the presence in the packaging medium of the high and medium OCL concentrations led to lower (*p* < 0.05) lipid oxidation development (fluorescent compound and TBARS detection, respectively). Furthermore, an increasing OCL presence led to an average decrease of peroxide and FFA content and to an average increase of the polyene index (PI). All OCL-packaged muscle showed lower average values of saturated fatty acids and ω3/ω6 ratio and higher average values of PI and monounsaturated fatty acid presence. This study provides a first approach to novel and beneficial use of the present marine waste to inhibit lipid damage of commercial canned fish.

## 1. Introduction

As a result of processing, the fishing, aquaculture, and foodstuff industries generate a wide range of byproducts and wastes [1,2]. Among such undervalued products, wastewaters generated by seafood processing have been recognised as rich in healthy and nutritional constituents but constitute one of the most important environmental problems of coastline areas [3,4]. Commonly, these byproducts are dumped into the sea without previous treatment of depuration, thus causing serious environmental pollution [3,5]. To prevent water pollution, achieve complete utilisation of available nutrients (mainly proteins, ω3 polyunsaturated fatty acids, and valuable flavour and aroma compounds), and offer a commercial gain for the food industry, an effective recycle and utilisation of wastewater byproducts is of critical importance [5,6].

On the basis of the great commercial importance of tuna species canning, the greatest efforts for employing wastewaters from seafood processing have been addressed for tuna cooking juices [7]. Thus, Ahn and Kim [8] showed that taurine, glutamic acid, phenylalanine, and alanine were the major amino acids resulting from neutrase hydrolysis of tuna skipjack (*Katsuwonus pelamis*) cooking juice. Later on, Jao and Ko [9] proved the DPPH (2,2′-diphenyl-1-picrylhydrazyl) radical scavenging capacity of the hydrolysate tuna (*Thunnus tonggol*) cooking juice by isolation and identification of the resulting peptides from proteolytic digestion. Remarkably, tuna cooking juices without previous enzymatic treatment were satisfactorily employed for producing dried tuna flavour powder [10] and proved to be rich in nutrients and antioxidant properties (DPPH and ABTS, 3-ethylbenzothiazoline-6-sulphonic acid, assays) [11].

Octopus species constitute popular seafood that are typically commercialised fresh, frozen, or dried salted, both at the artisan and industrial scales [12,13]. In addition to highly nutritional and medicinal value products, octopus processing has shown to generate a lot of byproducts including high functional value, thus indicating a promising potential for the integrated exploitation and utilisation of bioactive substances [14,15]. Among octopus processing wastes, cooking liquor or juice has acquired the interest of technologists and the fish trade, although previous research cannot be considered as abundant as in the case of tuna cooking liquor. Thus, Oh et al. [16] showed remarkable antihypertensive and antioxidant (Rancimat assay) effects for this kind of octopus waste material. Furthermore, Kim et al. [17] proved that a 70% ethanol extract from the cooking drip of Giant Pacific octopus (*Enteroctopus dofleini*) included a marked polyphenol compound content as well as manifested a radical scavenging activity (DPPH and FRAP, ferric reducing antioxidant power, assays) and an inhibitory activity against tyrosine and angiotensin I-converting enzyme. An antioxidant capacity (DPPH assay) was also detected by Choi et al. [18] in cooking drip from the same octopus species, with this effect increasing if a previous gamma-irradiation was applied. With an industrial and microbiological focus, Vázquez and Murado [19] showed that enzymatic hydrolysis of wastewater from industrial processing of octopus (*Octopus vulgaris*) could be a good source of peptones for lactic acid bacteria production.

In the current study, the preservative properties of waste liquor obtained from octopus (*Octopus vulgaris*) cooking were investigated. Three different volumes of such liquor (10, 15, and 50 mL) were included, respectively, in the packaging medium employed for Atlantic mackerel (*Scomber colias*) canning (C-10, C-15, and C-50 packaging conditions, respectively) and compared to control canned fish (C-CT packaging condition). The effect of OCL packaging on lipid hydrolysis and oxidation, fatty acid (FA) profile (saturated FA, STFA; monounsaturated FA, MUFA; polyunsaturated FA, PUFA), and FA ratios (polyene index, PI; ω3/ω6 ratio) in canned mackerel was determined.

## 2. Results and Discussion

### 2.1. Moisture and Lipid Content

Values obtained in raw samples for moisture (690.4 ± 19.1 g·kg^−1^ muscle) and lipid (80.7 ± 2.10 g·kg^−1^ muscle) constituents (Table 1) are in agreement with those values found for fatty fish species [20]. Canning process led to a decrease (*p* < 0.05) in moisture content, with values in the 596–611 g·kg^−1^ range. This level of decrease can be explained on the basis of denaturation and decrease of water-holding capacity of fish proteins as a result of the thermal treatment, thus leading to water loss from the muscle into the packaging medium [21,22]. Contrarily, lipid content in canned mackerel muscle showed a marked (*p* < 0.05) increase that can be explained as a result of the loss of water and hydrophilic constituents from the muscle into the packaging medium [23].

Concerning the effect of the OCL in the covering system, comparison of control canned fish with canned samples including the OCL packaging did not provide significant differences (*p* > 0.05) for moisture and lipid contents; additionally, a definite trend on the presence of both constituents was not detected (*p* > 0.05) by increasing or decreasing the OCL concentration tested. The fact that no differences of moisture value were detected can be explained on the basis that the canned fish muscle was imbibed in an aqueous packaging medium; therefore, a strong interchange of water between both phases included in the can would be expected to occur [23,24]. Concerning possible lipid content differences, relevant fish-to-fish differences have been reported for the lipid content of fish muscle as a result of several internal and external factors [20,25]; consequently, the OCL presence and concentration in the packaging medium would not be likely to produce content differences in this muscle constituent.

Previous research concerning the effect on moisture and lipid content in canned fish muscle as a result of including preservative compounds in the covering medium can be considered very scarce. According to the present study, no effect on lipid content in canned Chub mackerel (*S. colias*) was observed by including alga (*Fucus spiralis* or *Ulva lactuca*) extracts in the filling medium [23].

### 2.2. Determination of Lipid Hydrolysis

**Figure 1:**
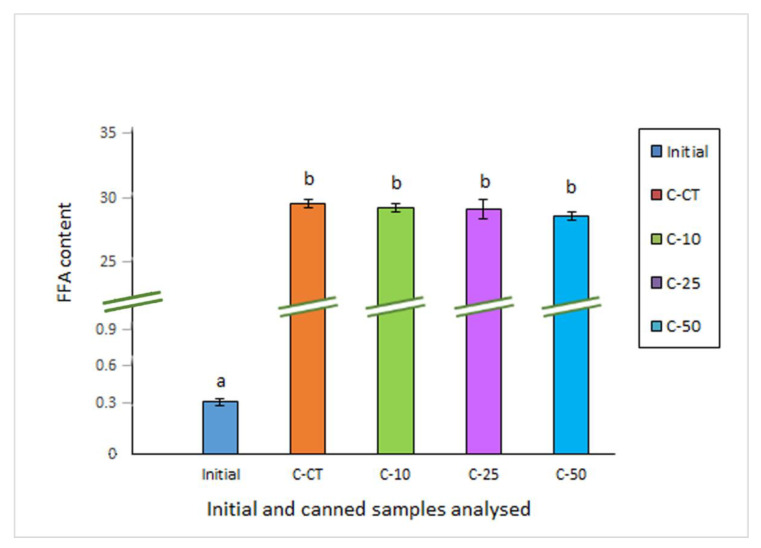
Determination of free fatty acid (FFA) content (g·kg^−1^ muscle) * in initial and canned mackerel packaged under different conditions including octopus cooking liquor (OCL) **. * Average values of four replicates (*n* = 4); standard deviations are indicated by bars. Average values accompanied by different lowercase letters (**a**,**b**) denote significant differences (*p* < 0.05) as a result of packaging condition. ** Packaging conditions as described in Table 1.

This damage pathway was analysed by the free FA (FFA) assessment. A very low value was detected in starting raw fish (Figure 1), thus showing a good quality of the starting material employed in the current study. Canning led to a great (*p* < 0.05) increase of FFA content in all kinds of canned fish (28.0–31.6 g·kg^−1^ muscle range) (Figure 1). This result was in agreement with previous research related to fish canning [26,27]. A lower average FFA content was detected in canned samples including OCL in the packaging medium when compared to their counterpart samples corresponding to the canned control; remarkably, decreasing average values were obtained by increasing the OCL presence in the covering medium.

It is generally accepted that accumulation of FFA resulting from lipid hydrolysis in fish muscle has no nutritional significance. Nevertheless, this damage pathway has been recognised as an important event during fish processing leading to deteriorative changes of muscle texture, acceleration of lipid oxidation compounds formation, and off-odour and off-taste development [28,29]. In the current research, FFA content can be considered as the result of several factors. First, the sterilisation process can lead to hydrolysis of lipid classes such as triacylglycerides (TG) and phospholipids (PL) [24]. On the other hand, FFA are known to be rapidly oxidised by heating according to the fact that they provide a greater accessibility to oxygen and other oxidants in general when compared to TG and PL [30]. Finally, preservative compounds (i.e., antioxidant molecules) present in the OCL-packaging medium may protect FFA from oxidation and subsequent breakdown during the heating process. According to the marked differences found between raw and all canned samples, a strong hydrolytic effect of heating on TG and PL was produced in the present study. Furthermore, none of the OCL concentrations tested led to significant differences (*p* > 0.05) when compared to the control packaging. However, the fact that a lower average FFA content was detected in canned fish by increasing the presence of OCL indicates that some inhibitory effect on FFA formation has been produced, this effect being more notorious in fish corresponding to the C-50 packaging condition.

Previous research provides contradictory results when addressing the effect of packaging conditions on lipid hydrolysis development during the canning process. Thus, Medina et al. [31] showed that the extent and mechanism of lipolysis were not influenced by the packaging medium used (brine and soybean oil) when considering canned albacore (*Thunnus alalunga*). However, hydrolytic rancidity showed that FFA content in canned silver carp *(Hypophthalmichthys molitrix*) including olive oil or sunflower oil as covering medium was lower than in the case of employing brine or soybean oil [32]. Furthermore, the use of sunflower oil increased the protective effect of processed tuna (*Thunnus albacares*) against lipid hydrolysis (FFA assessment) when compared to coconut and groundnut oils [33]. Remarkably, higher average FFA values were obtained in canned Atlantic mackerel (*Scomber scombrus*) by including *Bifurcaria bifurcata* extracts in the packaging medium [25]. Similarly, the presence of *F. spiralis* or *U. lactuca* extracts in the covering medium led to a higher average FFA value in canned Chub mackerel (*S. colias*) [23]. However, if a previous nine-day chilling storage was applied to canned Chub mackerel (*S. colias*), the presence of *F. spiralis* extracts in the packaging medium led to lower average FFA values in canned muscle [34].

### 2.3. Determination of Lipid Oxidation

Canning and thermal treatments in general have been proven to enhance lipid oxidation development during seafood processing [21,24]. This effect has been explained on the basis of the catalytic behaviour of thermal treatment on lipid oxidation development in marine lipids, such lipids being especially rich in PUFA. In order to provide an accurate analysis of the lipid oxidation evolution in the current study, determination of this damage pathway was carried out by assessing the formation of oxidation compounds at different levels, i.e., primary (peroxide value, PV), secondary (thiobarbituric acid reactive substances, TBARS), and tertiary (fluorescence ratio, FR).

Levels of primary oxidation compounds can be considered very low in raw and canned samples [35,36,37], with values in the 0.24–0.48 meq. active oxygen·kg^−1^ lipids range (Table 2). Notably, higher average values were detected in canned fish corresponding to OCL-packaging conditions when compared to canned control and initial fish. Thus, canned fish muscle including any of the OCL packaging conditions showed higher (*p* < 0.05) peroxide levels than the raw fish; among OCL-treated fish, the lowest average value was obtained in the C-50 batch.

As for peroxides, thiobarbituric acid index (TBA-i) levels observed in raw and canned samples can be considered low [33,35] and were in the 0.21–0.43 mg malondialdehyde·kg^−1^ muscle range. Additionally, TBARS formation during processing reflected a slight average value increase in all kinds of canned samples (Table 2). This increase was found to be higher (*p* < 0.05) in canned control samples, while canned samples including any of the OCL-packaging media did not provide significant differences (*p* > 0.05) with starting values. Remarkably, canned samples corresponding to C-25 condition showed lower values (*p* < 0.05) than their counterpart control samples.

Concerning tertiary lipid oxidation compounds, canning led to a general increase of fluorescent compound formation (FR increase), the highest average value being obtained for the control canned fish (Table 2). Notably, canned fish corresponding to the C-50 condition showed a significantly lower (*p* < 0.05) value than control canned fish and did not provide significant differences (*p* > 0.05) when compared to the starting raw fish. Consequently, an inhibitory effect on the formation of this kind of lipid oxidation molecules was detected in the C-50 packaging batch.

In the present study, heat treatment had two opposite effects on the different kinds of lipid oxidation compounds [38,39]. Thermal treatment oxidised and facilitated the formation of primary, secondary, and tertiary oxidation compounds. On the other hand, heat treatment itself may cause degradation of such molecules, especially those produced in the first stages (namely, peroxides) of the development of this damage pathway. At advanced stages of lipid oxidation, both peroxide and carbonyl compounds (i.e., TBARS) are susceptible to react with nucleophilic-type molecules present in the fish muscle and lead to fluorescent compound formation [24,40]. Concerning the effect of OCL presence in the packaging medium, a slight preservative effect on peroxide breakdown could be concluded in fish corresponding to all OCL-canning conditions. Related to secondary and tertiary oxidation compounds, a marked formation was detected in canned samples (comparison between raw and canned control samples), so that formation of such molecules can be considered more important than their breakdown. Notably, an inhibitory effect (*p* < 0.05) of OCL packaging could be concluded on TBARS and fluorescent compound formation in C-25 and C-50 batches, respectively.

An antioxidant behaviour of liquors obtained from octopus species cooking has already been mentioned in previous literature concerning in vitro assays. Thus, Oh et al. [16] studied the components of octopus cooking drips and proved an antioxidant behaviour according to the Rancimat assay. Similarly, cooking drip from Giant Pacific octopus (*E. dofleini*) showed an antioxidant capacity (DPPH assay) that could be increased by previous gamma-irradiation [18]. Kim et al. [17] studied the 70% ethanol extract from the same octopus species; notably, the radical scavenging activity (FRAP and DPPH assays) and the content on proteins and polyphenol compounds was enhanced by increasing the previous gamma-irradiation dose. Related to cooking juice of tuna species, Li et al. [11] demonstrated an antioxidant behaviour according to the DPPH and ABTS assays. Furthermore, Jao and Ko [9] isolated and identified seven antioxidant peptides from tuna (*T. tonggol*) cooking juice by reversed phase HPLC; peptide sequences comprised four to eight amino acid residues, including valine, serine, proline, histidine, alanine, asparagine, lysine, glutamic acid, glycine, or tyrosine. Interestingly, low-molecular-weight peptides have been reported as having antioxidant properties (i.e., free radical scavengers and reducing agents) [41,42].

Previous research has addressed the effect on lipid oxidation by including other sources of antioxidant compounds in the packaging medium during fish canning. Thus, packaging employing extra-virgin olive oil showed a marked inhibition of lipid oxidation progress in canned tuna (*T. alalunga*) when compared to a covering medium consisting of a brine solution [43]; this effect was attributed to the high presence of polyphenol compounds in extra-virgin olive oil. A lower development of lipid oxidation (i.e., fluorescent compound formation) was observed by Naseri and Rezaei [44] in sunflower oil-canned sprat (*Clupeonella cultriventris*) when compared to its counterpart packaged in brine solution. Furthermore, the employment of olive oil as filling medium led to a lower TBARS formation in canned silver carp (*H. molitrix*) than in the case of including sunflower oil, soybean oil, or brine as packaging medium [31]. Similarly, the use of sunflower oil increased the protective effect of canned tuna (*T. albacares*) against lipid oxidation (TBARS assessment) when compared to packaging with coconut and groundnut oils [32]. The use of packaging media including antioxidant compounds obtained from algae species has also proved the inhibition of lipid oxidation progress in canned fish. This result was obtained in canned Atlantic mackerel (*S. scombrus*) by employing *B. bifurcata* extracts [33] and in Chub mackerel (*S. colias*) by addition of *F. spiralis* or *U. lactuca* extracts [23].

### 2.4. FA Analysis

Initial mackerel presented the following FA average composition (g·kg^−1^ lipids): 30.5 (C14:0), 4.5 (C15:0), 133.0 (C16:0), 37.8 (C16:1ω7), 7.3 (C17:0), 39.0 (C18:0), 162.3 (C18:1ω9), 35.8 (C18:1ω7), 8.5 (C18:2ω6), 20.9 (C20:1ω9), 2.7 (C20:2ω6), 7.3 (C20:4ω6), 3.5 (C22:1ω9), 62.2 (C20:5ω3), 2.9 (C22:4ω6), 5.1 (C24:1ω9), 13.4 (C22:5ω3), and 124.8 (C22:6ω3). FA analysis was carried out on all canned samples (data not shown). In order to better focus on possible quality changes, discussion of FA results is addressed to FA groups (STFA, MUFA, and PUFA) and FA ratios (PI and ω3/ω6).

Concerning the STFA group, comparison between initial and control canned samples showed a slight increase of the average value as a result of the canning process (Table 3); remarkably, lower average values were detected in all canned samples including OCL in the packaging medium. However, differences were only found significant (*p* < 0.05) by comparing the initial samples and canned fish corresponding to C-25 and C-50 packaging conditions.

The MUFA average content in fish muscle showed a general decrease after the canning process (Table 3); however, the presence of OCL in the packaging medium led to higher average values than in the control canned fish. Differences were found significant (*p* < 0.05) in the case of the C-25 batch, with such samples showing a lower (*p* < 0.05) MUFA presence than in the initial raw fish.

An important increase in PUFA presence was detected in all canned samples when compared with the initial fish (Table 3); this effect was found to be significant (*p* < 0.05) in the case of C-25 canned fish. Notably, canned fish corresponding to C-25 and C-50 batches provided higher average values than fish corresponding to the control canned batch. A PUFA retention was also detected in canned salmon (*Salmo salar*) when including seaweed (*U. lactuca, Durvillaea antartica,* and *Pyropia columbina*) extracts as part of the covering liquid [37]; additionally, a higher retention of astaxanthine was detected in canned salmon by the presence of algae extracts.

**Figure 2:**
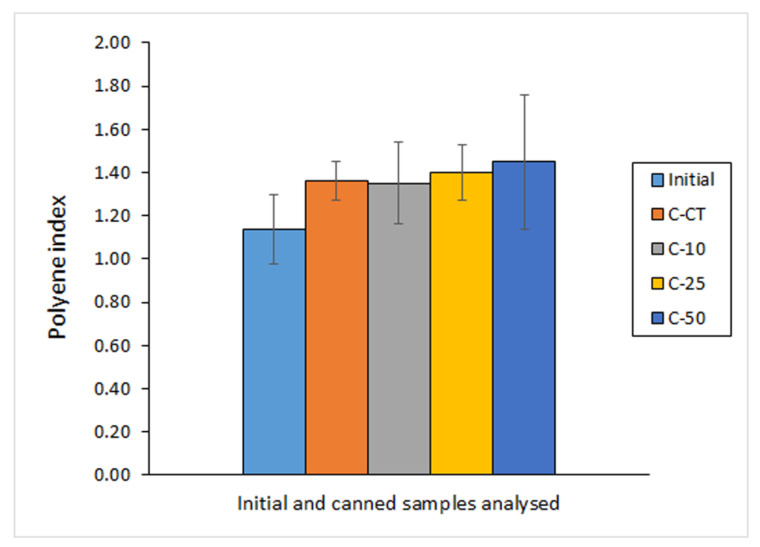
Determination of the polyene index * in initial and canned mackerel packaged under different conditions including octopus cooking liquor (OCL) **. * Average values of four replicates (*n* = 4); standard deviations are indicated by bars. ** Packaging conditions as described in Table 1.

The assessment of the PI, measured as a FA content ratio, has recently attracted a great attention as a way of measuring the possible increase or decrease of the PUFA content during fish canning or fish processing in general and being directly related to the nutritional value [38,39]. In the current study, a general increase of the average PI score was obtained after the canning process (Figure 2). This increase was found to be higher when increasing the presence of OCL in the packaging medium; remarkably, the highest average value was obtained in fish corresponding to the C-50 batch. Therefore, some preservative effect on PUFA compounds could be inferred from the presence of OCL in the covering medium.

Previous research related to the addition of antioxidant compounds in the packaging medium has already shown a preservative effect on the PI. Thus, Ortiz et al. [37] showed a significant PI retention in canned Atlantic salmon (*S. salar*) muscle when packaged in a water medium including an ulte (basal part of alga *D. antarctica*) extract; however, no differences were obtained in such study when other algae (cochayuyo, frond of *D. antarctica*; *U. lactuca*; *p. columbina*) extracts were included in the water-packaging systems. Similarly, higher PI scores were observed in Atlantic mackerel (*S. scombrus*) by addition of *B. bifurcata* extracts [25] and in Chub mackerel (*S. colias*) by addition of *F. spiralis* or *U. lactuca* extracts [23]. Contrarily, no effect on the PI of a three-year canned sprat (*C. cultriventris*) was observed with brine as packaging medium instead of sunflower oil [44].

Concerning PUFA series (namely, ω3 and ω6), great attention has recently been accorded to the ω3/ω6 ratio in seafood and food in general [45]. In order to prevent inflammatory, cardiovascular, and neurological disorders, the World Health Organisation (WHO) currently recommends that this ratio should be higher than 0.1 in the human diet [46]. Additionally, the European Nutritional Society reported that a human diet with a ω3/ω6 ratio of 1/5 or higher would have health benefits [47].

In the current study, ω3/ω6 ratio of raw and canned samples was included in a straight range (i.e., 9.0–10.8) (Table 3). Notably, the canning process led to a significant (*p* < 0.05) increase (comparison between initial and control canned samples); however, the presence of OCL in the packaging medium led to lower average values than in control canned fish, although significant differences were not attained (*p* > 0.05). Among OCL-treated fish, the highest average values were observed in mackerel corresponding to C-50 batch. Nevertheless, values in all cases can be considered as highly nutritional, according to the abovementioned health requirements for this FA ratio.

## 3. Materials and Methods

### 3.1. Sample Preparation

Commercial OCL was provided by Frigoríficos Rosa de los Vientos S. L. (Marín, Pontevedra, Spain) in vacuum-sealed bottles protected from light. Liquor was stored under refrigerated conditions (4 °C) before use.

Sample (40 fish) of Atlantic Chub mackerel (*S. colias*) (length range: 29–35 cm; weight range: 240–300 g) were obtained at Vigo harbour (North-Western Spain) in May 2021 and transported (10 min) on ice to the laboratory. As the initial fish batch (starting raw mackerel; raw control), eight fish were selected and divided into four groups (two fish per group). Sample were beheaded, eviscerated, filleted, and analysed for moisture and lipid content, lipid damage, and FA composition according to the methods described later. Within each group, the white muscle was analysed independently (*n* = 4).

The remaining samples (32 fish) were stored at –40 °C for 48 h and then kept frozen (–18 °C) for a 6-month period. At that time, fish were thawed overnight (4 °C), beheaded, eviscerated, and filleted. Then, 45-g portions of mackerel fillets were placed in small flat rectangular cans (105 × 60 × 25 mm; 150 mL). As packaging media, 10, 25, and 50 mL of OCL were added to the cans, followed by the addition of distilled water (85, 70, and 45 mL, respectively) to be filled. As a result, low-concentrated (C-10 batch), medium-concentrated (C-25 batch), and high-concentrated (C-50 batch) packaging conditions were prepared, respectively. Additionally, canned control samples were prepared by introducing in the can 45 g of fish fillet and filling it with distilled water (95 mL; C-CT batch). The various OCL concentrations used in this study were based on several preliminary tests. Thus, a 50-mL volume addition corresponded to the highest concentration without modifying the sensory descriptors of canned mackerel (i.e., flesh colour, odour, or flavour). In order to analyse the effect of the OCL content, two lower volumes (namely, 10 and 25 mL, C-10 and C-25 batches, respectively) were also checked in this study.

Each can was prepared with a single-fish fillet. For each packaging condition, eight different cans were prepared that were pooled into four groups (two cans per group), each group being analysed independently (*n* = 4). All cans were vacuum sealed in a horizontal steam retort (115 °C, 45 min; *F*_o_ = 7 min) (CIFP Coroso, Ribeira, A Coruña, Spain). Once the heating time was completed, steam was cut off and air was used to flush away the remaining steam. The cans were cooled at reduced pressure. After a 3-month storage at room temperature (20 °C), the cans were opened, and the liquid part was carefully drained off gravimetrically and filtered through a filter paper. Then, the mackerel white muscle was separated, wrapped in filter paper, and used for analysis.

All solvents and chemical reagents used were of reagent grade (Merck, Darmstadt, Germany).

### 3.2. Determination of Moisture and Lipid Content

Moisture content was determined as the weight difference in homogenised fish white muscle (1–2 g) before and after 4 h at 105 °C [48]. Results were calculated as g·kg^−1^ muscle.

Lipids were obtained by extraction of the mackerel white muscle by applying the Bligh and Dyer [49] method, which employs a chloroform-methanol (1:1) mixture. Quantification was carried out according to Herbes and Allen [50]. Lipid content was calculated as g·kg^−1^ mackerel muscle.

### 3.3. Assessment of Lipid Damage

FFA content was determined on the lipid extract of the fish muscle by the Lowry and Tinsley [51] method, which is based on complex formation with cupric acetate-pyridine followed by spectrophotometric (715 nm) assessment (Beckman Coulter DU 640 spectrophotometer, Beckman Coulter Inc., Brea, CA, USA). Results were calculated as g FFA·kg^−1^ muscle.

PV was determined spectrophotometrically (520 nm) on the lipid extract by peroxide reduction with ferric thiocyanate [52]. Results were calculated as meq. active oxygen·kg^−1^ lipids.

TBA-i was determined according to Vyncke [53]. Content of TBARS was spectrophotometrically measured at 532 nm and calculated from a standard curve using 1,1,3,3-tetraethoxy-propane (TEP). Results were calculated as mg malondialdehyde·kg^−1^ muscle.

The formation of fluorescent compounds (Fluorimeter LS 45; Perkin Elmer España; Tres Cantos, Madrid, Spain) was determined in the lipid extract of the fish muscle as described previously [39]. The relative fluorescence (RF) was calculated as follows: RF = *F/F_st_*, where *F* is the fluorescence measured at each excitation/emission wavelength pair and *F_st_* is the fluorescence intensity of a quinine sulphate solution (1 µg·mL^−1^ in 0.05 M H_2_SO_4_) at the corresponding wavelength pair. Results were calculated as the FR, which was calculated as the ratio between the two RF values: FR = RF_393/463 nm_/RF_327/415 nm_.

Lipid extracts were converted into FA methyl esters (FAME) by using acetyl chloride in methanol and then analysed using a Perkin-Elmer 8700 gas chromatograph (Madrid, Spain) equipped with a fused silica capillary column SP-2330 (0.25 mm i.d. × 30 m, 0.20 μm film, Supelco Inc., Bellefonte, PA, USA) [12]. Peaks corresponding to FAME were identified by comparing their retention times with those of standard mixtures (Qualmix Fish, Larodan, Malmo, Sweden; FAME mix, Supelco, Inc.). Peak areas were automatically integrated; C19:0 FA was used as the internal standard for quantitative purposes.

Content of each FA was calculated as g·100 g^−1^ total FA. Such values were employed in order to obtain the content on FA groups (STFA, MUFA, and PUFA) (g·100 g^−1^ total FA) and the ω3/ω6 ratio. Additionally, the PI was calculated as the following FA content ratio: (C20:5ω3 + C22:6ω3)/C16:0.

### 3.4. Statistical Analysis

Chemical values were subjected to the ANOVA method to explore differences obtained from the effect of canning and packaging conditions. As expressed above, four replicates (*n* = 4) were considered in the study. The least-squares difference (LSD) method was used to perform the comparison of means. Analyses were carried out using the PASW Statistics 18 software for Windows (SPSS Inc., Chicago, IL, USA); differences were considered significant for a confidence interval at the 95% level (*p* < 0.05).

## 4. Conclusions

The preservative properties of waste liquor obtained from common octopus cooking were investigated. OCL was included in the aqueous packaging medium employed for mackerel canning. As a result, the canning process led to an increase of lipid content, lipid oxidation (development of fluorescent compounds), and hydrolysis (FFA content increase) and ω3/ω6 ratio in fish muscle. In all canned samples, primary (peroxides) and secondary (TBARS) levels of lipid oxidation were low (0.29–0.48 meq. active oxygen·kg^−1^ lipids and 0.26–0.43 mg malondialdehyde·kg^−1^ muscle, respectively). Remarkably, the presence in the packaging medium of OCL led to lower (*p* < 0.05) TBARS (0.26 mg malondialdehyde·kg^−1^ muscle) and fluorescent compound (3.72) formation in canned fish corresponding to C-25 and C-50 batches, respectively. Furthermore, an increasing OCL presence led to an average decrease of peroxide and FFA content and to an average increase of the PI. All OCL-packaged muscle showed lower average values of STFA and ω3/ω6 ratio and higher average values of PI and MUFA presence. Among OCL-treated fish, the C-50 batch showed the lowest average peroxide (0.31 meq. active oxygen·kg^−1^ lipids) and FFA (27.98 g·kg^−1^ muscle) values and the highest PI scores (1.45). Globally, this concentration is considered the most accurate for quality enhancement in the present investigation.

The current study constitutes a novel and beneficial strategy to enhance the quality of commercial canned fish, thus enabling environmental sustainability and circular economy. OCL was satisfactorily employed for quality enhancement of a canned product. It is considered that the development of optimised conditions of this cooking-liquor packaging system may open the way to its application in all kinds of canned fish species, including high-value fatty fish such as tuna, bonito, or salmon. Further research envisaged to analyse molecules present in OCL and involved in the mechanism of lipid preservation ought to be addressed.

## Figures and Tables

**Table 1:** Moisture and lipid content (g·kg^−1^ muscle) * in initial and canned mackerel packaged under different conditions including octopus cooking liquor (OCL) **.

Constituent	Initial Fish	Canned Fish			
		C-CT	C-10	C-25	C-50
Moisture	690.4 ^b^ (19.1)	596.6 ^a^ (17.1)	601.0 ^a^ (20.9)	610.6 ^a^ (24.3)	598.5 ^a^ (30.8)
Lipids	80.7 ^a^ (21.0)	153.5 ^b^ (9.5)	148.4 ^b^ (20.5)	132.6 ^b^ (12.6)	156.2 ^b^ (34.5)

* Average values of four replicates (*n* = 4); standard deviations are indicated in brackets. Average values accompanied by different lowercase letters (^a, b^) denote significant differences (*p* < 0.05) as a result of packaging condition. ** Packaging conditions: C-CT (control packaging), C-10 (low-concentrated OCL packaging), C-25 (medium-concentrated OCL packaging), and C-50 (high-concentrated OCL packaging).

**Table 2:** Determination of lipid oxidation * in initial and canned mackerel packaged under different conditions including octopus cooking liquor (OCL) **.

Quality Index	Initial Fish	Canned Fish			
		C-CT	C-10	C-25	C-50
Peroxide value (meq. active oxygen·kg^−1^ lipids)	0.24 ^a^ (0.04)	0.29 ^ab^ (0.08)	0.48 ^b^ (0.17)	0.35 ^b^ (0.03)	0.31 ^b^ (0.09)
Thiobarbituric acid index (mg malondialdehyde·kg^−1^ muscle)	0.21 ^a^ (0.07)	0.43 ^b^ (0.10)	0.42 ^ab^ (0.13)	0.26 ^a^ (0.03)	0.29 ^ab^ (0.11)
Fluorescence ratio	3.53 ^a^ (0.47)	4.80 ^b^ (0.34)	4.18 ^ab^ (0.92)	3.87 ^ab^ (0.80)	3.72 ^a^ (0.66)

* Average values of four replicates (*n* = 4); standard deviations are indicated in brackets. Average values accompanied by different lowercase letters (^a, b^) denote significant differences (*p* < 0.05) as a result of packaging condition. ** Packaging conditions as described in Table 1.

**Table 3:** Values * obtained for fatty acid (FA) groups (g·100 g^−1^ total FA) and total ω3/total ω6 ratio in initial and canned mackerel packaged under different conditions including octopus cooking liquor (OCL) **.

FA Group or Ratio	Initial Fish	Canned Fish			
		C-CT	C-10	C-25	C-50
Total saturated FA	33.79 ^b^ (0.34)	34.41 ^ab^ (2.81)	32.16 ^ab^ (1.49)	33.18 ^a^ (0.18)	32.51 ^a^ (1.24)
Total monounsaturated FA	35.35 ^b^ (2.82)	30.51 ^ab^ (6.44)	34.79 ^ab^ (0.95)	31.29 ^a^ (0.95)	32.36 ^ab^ (4.16)
Total polyunsaturated FA	30.36 ^a^ (3.04)	35.07 ^ab^ (3.79)	33.05 ^ab^ (2.35)	35.52 ^b^ (1.13)	35.12 ^ab^ (4.87)
ω3/ω6 ratio	9.04 ^a^ (1.10)	10.83 ^b^ (0.46)	9.09 ^ab^ (1.86)	9.53 ^ab^ (0.67)	9.86 ^ab^ (1.63)

* Average values of four replicates (*n* = 4); standard deviations are indicated in brackets. Average values accompanied by different lowercase letters (^a, b^) denote significant differences (*p* < 0.05) as a result of packaging condition. ** Packaging conditions as described in Table 1.

## Data Availability

The data presented in this study are available on request from the corresponding author.

## Sample Availability

Samples of the compounds are not available from the authors.
